# The Fabrication of Au@C Core/Shell Nanoparticles by Laser Ablation in Solutions and Their Enhancements to a Gas Sensor

**DOI:** 10.3390/mi9060278

**Published:** 2018-06-01

**Authors:** Xiaoxia Xu, Lei Gao, Guotao Duan

**Affiliations:** Key Laboratory of Materials Physics, Anhui Key Laboratory of Nanomaterials and Nanotechnology, Institute of Solid State Physics, Chinese Academy of Sciences, Hefei 230031, China; xxxu@issp.ac.cn (X.X.); lgao@issp.ac.cn (L.G.)

**Keywords:** laser ablation, core/shell nanostructure, ultrathin carbon layer, gas sensing

## Abstract

A convenient and flexible route is presented to fabricate gold noble metal nanoparticles wrapped with a controllable ultrathin carbon layer (Au@C) in one step based on laser ablation of the noble metal targets in toluene-ethanol mixed solutions. The obtained metal nanoparticles were <20 nm in size after ablation, and the thickness of the wrapped ultrathin carbon layer was 2 nm in a typical reaction. The size of the inner noble metal nanoparticles could be controlled by adjusting the power of laser ablation, and the thickness of the ultrathin carbon layer can be controlled from 0.6 to 2 nm by laser ablation in different components of organic solution. Then the resultant Au@C core/shell nanoparticles were modified on the surface of In_2_O_3_ films through a sol-gel technique, and the hydrogen sulfide (H_2_S) gas-sensing characteristics of the products were examined. Compared to pure and Au-modified In_2_O_3_, the Au@C-modified In_2_O_3_ materials exhibited a revertible and reproducible performance with good sensitivity and very low response times (few seconds) for H_2_S gas with a concentrations of 1 to 5 ppm at room temperature. Evidence proved that the ultrathin carbon layer played an important role in the improved H_2_S sensor performance. Other noble metals wrapped by the homogeneous carbon shell, such as Ag@C, could also be prepared with this method.

## 1. Introduction

Core/shell nanostructure nanocomposites, in which the inner nanoparticle is encapsulated and protected against agglomeration, adsorption, or chemical reaction by an outer shell, have attracted much attention due to their fantastic physical, chemical, biological, and catalytic properties [1,2,3]. The thickness of the outer shell plays a significant role in the performance of the nanocomposites. For example, the surface enhanced Raman scattering (SERS) of noble metal nanomaterials coated with special materials shell to detect some target molecules need the outer shell in nanometer scale, and the SERS signal will be weakened, or disappear, if the coating shell is too thick [4]. On the other hand, graphene or graphene-like structure materials process unique chemical and physical properties and have become a hot research topic since they appeared. As a sheath coating on nanoparticles they could effectively enhance the properties of the nanoparticles [5], and it requires the thickness of the outer carbon shell with one or several layers. Now there are even many methods to coat the nanoparticles, such as vapor deposition [6,7,8], solution dipping [9,10], sol-gel coating, and so on [11]. However, it is still a challenge to obtain the nanoparticles wrapped with ultrathin and homogeneous outer layers with the above methods, especially for the noble metal nanoparticles.

Laser ablation of the metal targets in liquids could easily and effectively prepare such structure materials in only one step [12,13,14]. Compared with the conventional method, it is an attractive green and versatile technique to prepare various metal nanoparticles, such as Au, Ag, Pt, and Ni, etc. The metal nanoparticles obtained by this method have excellent chemistry, metastable composition, easily functionalizable surfaces, high purity, and good dispersity, etc., and these properties are closely related to the applications such as catalysis and SERS research [13,15]. For example, using 532 nm output from a pulsed Nd:YAG laser (10 ns, 10 Hz) Shaji et al. successfully obtained ZnO nanoparticles with zinc metal as the target in distilled water at different water temperatures, and found that the morphology, structure, chemical state and optical properties of ZnO nanoparticles were closely related with the temperature and laser fluence [16]. Kautek et al. prepared Ni/NiO_x_ core/shell nanoparticles in water and alcoholic fluids, and the nature of the fluid, the laser fluence, and the number of laser pulses decided the size distribution of the products. Through the changes of these parameters, the size distribution of Ni/NiO_x_ core/shell nanoparticles was changed from 10 to 30 nm [17]. If only organic solvents are chosen as the liquids, it will be an ideal carbon source to form ultrathin carbon layers. By two sequential processes during ablation, i.e., formation of the noble metal nanoparticles and subsequent carbon-deposition, the homogeneous and ultrathin carbon layer-wrapped noble metal nanoparticles will be obtained in one step with this method. The production with a unique structure will be an effective and excellent modifying material for application of a semiconductor metal oxide-based gas sensor.

Semiconductor metal oxide-based gas sensors have received extensive research because they can detect the toxic, harmful, inflammable, or explosive gases quickly, efficiently, and accurately [18,19,20,21]. Among varieties of sensitive materials, indium oxide (In_2_O_3_) is a promising material for semiconductor gas sensor due to its peculiar properties, such as a wide band gap (3.56 eV), low resistance, and good catalysis [22,23,24]. Up to now, In_2_O_3_ has been widely applied to detect H_2_, CO, O_3_, and volatile organic compounds, etc. It is well known that the gas-sensing mechanism of semiconductors is based on the oxidation-reduction reaction between the surface of the sensor material and the test gas [25,26]. In order to improve the gas response and selectivity of semiconductor oxides, a nanoparticle-modifying method is often adopted, especially noble metal nanoparticles [27]. The reason is that it can easily change the electronic structure or space charge layer thickness of sensing films and improve the gas sensing performance. Herein, we report that modifying Au and Au@C nanoparticles prepared by laser ablation in liquid onto In_2_O_3_ film can lead to a greatly enhanced sensing sensitivity to H_2_S at room temperature.

## 2. Experimental Section

### 2.1. Au and Au@C Colloidal Solution Preparation

Typically, a gold (or silver) plate (99.99%, 1.5 × 1 cm^2^, purchased from Sinopharm Chemical Reagent Limited Corporation, Shanghai, China) is fixed on a bracket in a quartz glass vessel and immersed in 10 mL toluene-ethanol mixed solution with a volume ratio of 9:1. The vessel was placed on a horizontal platform. The plate in the solution was irradiated, while the solution was continuously stirred, by the first harmonic of a Nd:YAG (yttrium aluminum garnet) laser (1064 nm in wavelength, 10 Hz in frequency, and 10 ns in pulse duration) with the power of 60 mJ/pulse and the spot size of about 2 mm in diameter on the plate. A purple colloidal solution was formed during the irradiation for 20 min. The ablation of the target in water was also carried out for reference. After ablation, the solution was centrifuged at 12,000 rpm, and the obtained Au@C colloidal solution was rinsed several times with ethanol in order to remove the residual toluene and dehydrated at 60 °C for 12 h. The Au colloidal solution prepared in water was centrifuged under the same condition and Au nanoparticles were dispersed in 2 mL ethanol.

### 2.2. Preparation of Au or Au@C Modified In_2_O_3_ Film-Based Sensing Devices

Firstly, 1.0 g indium nitrate hydrate powder was dispersed in 80 mL deionized water with continual stirring by a magnetic rotor for 10 min. Subsequently, 0.6 g urea was added to the above colorless precursor solution and stirred for 20 min to obtain a mixture solution. Secondly, a 2D ordered polystyrene spheres (PS) colloidal monolayer template on a glass slide with the sphere diameter of 1000 nm was prepared by air/water interfacial assembly. Then the PS monolayer colloidal template on the glass slide was peeled off and floated onto the surface of pure water in a beaker due to surface tension of the solution. A ceramic tube was used to pick up the PS monolayer colloidal template from the bottom of liquid surface and dried at 80 °C for 2 h. Thirdly, the ceramic tube coated by the PS monolayer colloidal template was impregnated to the above mixture solution and transferred into a sealed stainless steel reactor and kept at 90 °C for 2 h. After the reaction the ceramic tube was washed by deionized water three times to remove the excess mixture solution. Fourthly, the ceramic tube was heated at 300 °C for 2 h to burn the PS template away and the In_2_O_3_ thin film was formed on the tube. Finally, Au and Au@C nanoparticles were modified onto the In_2_O_3_ thin film by impregnating it in the Au and Au@C colloidal ethanol solution and dried at 60 °C for 1 h. Thus, the Au- or Au@C-modified In_2_O_3_ film-based sensing devices were fabricated.

### 2.3. The Gas Sensing Measurements and Characterization Methods

All the gas sensing performances were measured in a static system with a volume of 20 L by the measurement of electric circuit at room temperature, in which a fixed resistor with the values ranging from 0.1 to 100 M ohm were connected in series on a circuit board to adjust the voltage of the sensor. This system was consisted by several sections such as manometers, electromagnetic cut-off valves, hydrometer, measuring chamber, gas pressure stabilizers and the signal processing system. Agilent U8002A DC power (San Jose, CA, USA) provided a 10 V regulated power supply, and Agilent mod. U3606A (San Jose, CA, USA) collected and recorded the voltage change on the fixed resistor by the computer. The H_2_S gas with required quantities was injected into the measure system by a syringe, which was calculated by the ideal gas equation of state from the pristine gas cylinders of 10,000 ppm obtained commercially. Firstly the pure air was introduced into the measure system for 30 min and guaranteed the sensor’s ambience with 60% RH, then a concentration of diluted H_2_S gas was injected and kept for 30 s, and the corresponding electric response signal of gas sensor was recorded by computer. Then the pure air was introduced into the measure system again to remove the H_2_S gas and after 60 s another concentration of diluted H_2_S gas was injected and kept for 30 s again, then cycled the above operation. In order to evaluate the selectivity performances of the sensor, some oxidizing or reducing gases such as C_3_H_6_O, H_2_, C_2_H_6_O, NH_3_, CH_4_, and SO_2_ were injected into the measurement system. A vaporizer was used to control the environmental humidity in the system, and a lab-made software was used to control the heating voltage and record data.

The resultant products were characterized by a field emission scanning electron microscope (FE-SEM, Hitachi, SU8020, Tokyo, Japan) and a transmission electron microscope (TEM, JEM-200CX, Tokyo, Japan). The optical absorption spectra were measured on a Cary-5E UV–VIS–NIR spectrophotometer (San Jose, CA, USA). The Raman spectra were recorded on a microscopic confocal Raman spectrometer (Renishaw inVia Reflex, London, UK) using a laser beam of 532 nm wavelength, 1 mW power and 5 μm spot size on the sample area. The gravimetric analysis was measured on TGA Q5000IR (Cranston, RI, USA).

## 3. Results and Discussion

**Figure 1:**
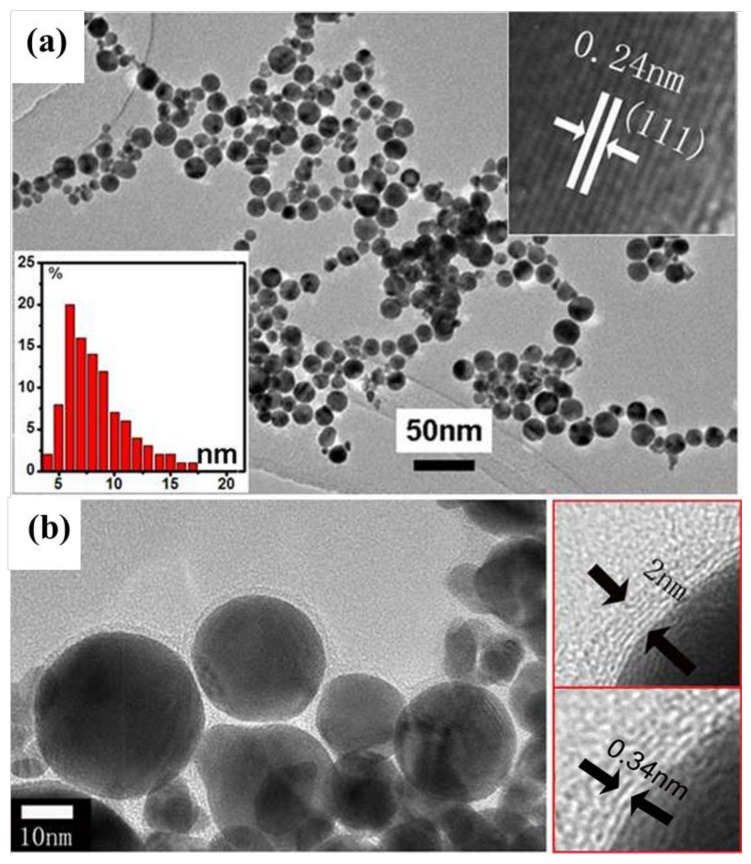
The TEM examination of the products induced by laser ablation of Au target in toluene-ethanol solution with the volume ratio 9:1 and the power of 60 mJ/pulse. (**a**): TEM image at low magnification. Insets: particle size distribution (lower-left) and high resolution TEM image of a partial particle showing Au (111) plane fringe (upper-right); and (**b**) local magnified image of (**a**). The insets show the thickness of the ultrathin carbon layer (upper-right) and the fringe spacing in the shell layer (lower-right).

In a typical reaction, using a toluene-ethanol mixed solution with a volume ratio of 9:1 as the laser ablation liquid, Au nanoparticles wrapped with an ultrathin carbon layer (Au@C) are obtained. The morphology and structure of the products are measured by TEM, as shown in Figure 1. It can be seen that the inner Au nanoparticles are spherical in structure with a diameter in the range of 5 to 15 nm and a mean size of 12 nm. Figure 1b is a larger image of Figure 1a, and the particles are much larger than the ones shown in Figure 1a. From the high-resolution TEM image of a partial particle, the clear crystal lattice can be discovered with a spacing of the lattice fringe of 0.24 nm, corresponding to the (111) plane of Au. Such a higher crystal surface index can ensure the prepared material with a better catalytic activity. The outer ultrathin carbon layer is about 2 nm in thickness with a spacing of the lattice fringe of 0.34 nm, which corresponds to the (001) plane of carbon. In addition, the energy spectrum of Au@C nanoparticles is also shown in the Figure S1, and it can be seen that the Au nanoparticles are indeed coated by a carbon layer, which corresponds to the results of high-resolution transmission electron microscopy (HRTEM). At the same conditions, using water solution as laser ablation liquids only Au nanoparticles with a size below 20 nm are formed after laser ablation of the Au target, and no carbon shell appears, as shown in Figure S2. Using water solution as laser ablation liquids only Ag nanoparticles without carbon shell is prepared with a size of about 50 nm after laser ablation of Ag target shown in Figure S3. Similarly, ablation of Ag target in the toluene-ethanol mixed solution with the same volume ratio leads to the wrapped Ag nanoparticles (Ag@C) with the average size of 15 nm, as shown in Figure S4. In addition, the energy spectrum of Ag@C nanoparticles is also shown in the Figure S5, which indicates that the Ag@C samples have been prepared successfully by the same method. The coating layer is also about 2 nm in thickness. In order to assess the amount of carbon in the shells of Au@C nanoparticles, a gravimetric analysis is measured and the results can be seen in the Figure S6. It can be seen that there is a mass loss of 0.94% at first, which corresponds to the small adsorbed molecules in the samples such as the water molecules. Then the other mass loss occurs from 199.7 °C, which corresponds to the combustion of the carbon shells. This indicates that the amount of carbon in the shells of Au@C nanoparticles is about 3.96%. The selected area electronic diffraction of Ag@C illustrated that the inner Ag nanoparticles have a good crystallinity. With this method, other types of metal nanoparticles wrapped with ultrathin carbon layers may also be obtained.

**Figure 2:**
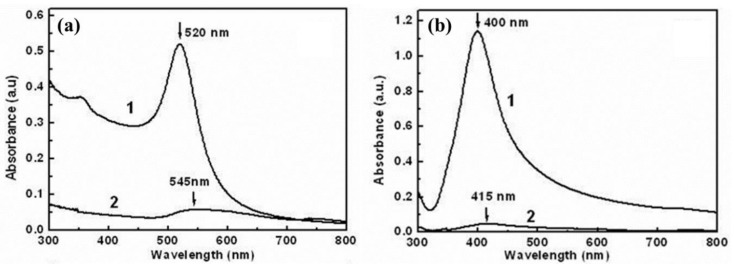
Optical absorbance spectra of the colloidal solutions induced by ablation of (**a**) Au target and (**b**) Ag target in solutions. Curve (1): ablation in water; and curve (2): ablation in the toluene-ethanol mixed solution with the volume ratio 9:1.

The optical absorbance spectra of Au and Au@C colloidal solutions are shown in Figure 2a. In the measurement of optical absorbance spectra of all the samples, which are prepared at the same conditions with the power of 60 mJ/pulse and the spot size of about 2 mm in diameter on the plate for 20 min, 3 mL sample water solution (0.1 g/L) is put into a quartz cell with 12.5 × 12.5 × 45 mm^3^ and the same quartz cell with 3 mL pure water solution is used as a reference. Then they are measured with a wavelength of 300 to 800 nm. For ablation of the Au target in water, there exists an absorption band around 520 nm, which corresponds to the well-known surface plasmon resonance (SPR) of Au nanoparticles, indicating formation of Au nanoparticles in the water [28,29,30,31]. However, ablation in the toluene-ethanol solution only leads to a weak and broad absorption band around 550 nm, as shown in Figure 2a. Similarly, for Ag, there is a strong SPR of Ag nanoparticles around 400 nm after ablation in water, and a very small and broad absorption band around 415 nm for ablation in the mixed solution, as illustrated in Figure 2b. Obviously, ablation in the mixed solution induces the red-shift and significant decrease of the optical absorption band for the Au@C and Ag@C samples.

**Figure 3:**
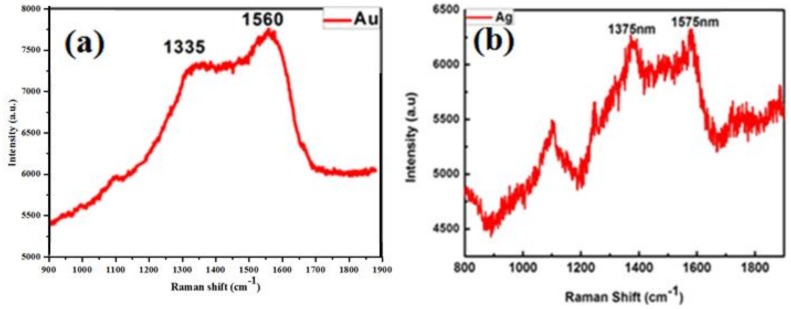
Raman spectrum for (**a**) Au@C colloidal solutions; and (**b**) Ag@C colloidal solutions.

In order to further reveal the structural information about the carbon coating layer surrounding the Au nanoparticles, Raman spectral measurement is conducted for the carbon wrapped Au nanoparticles, and the result is shown in Figure 3a. It can be found that there are two broad peaks around 1350 cm^−1^ and 1570 cm^−1^, correspond to D band (1355 cm^−1^) and G band (1590 cm^−1^) of graphitic carbon, respectively [32,33]. For the carbon-wrapped Ag nanoparticles, the Raman spectrum is also similar, as shown in Figure 3b. Obviously, it can indicate that the outer shell is ultrathin carbon layer, corresponding to the results of TEM. Thus, the noble metal nanoparticles wrapped with controllable ultrathin carbon layer are fabricated by one-step based on laser ablation in ethanol-toluene mixed solutions.

**Figure 4:**
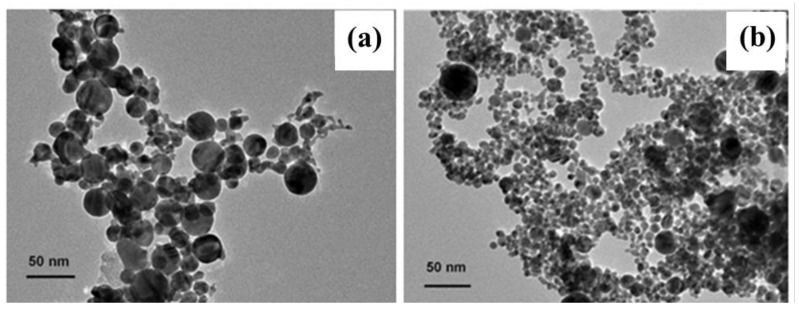
The TEM morphology characterization of the Au@C nanoparticles induced by the different ablation power. (**a**) 100 mJ/pulse; and (**b**) 40 mJ/pulse.

In addition, the size of the inner noble metal nanoparticles can be controlled by the ablation power. Taking the Au@C nanoparticles as an example, the nanoparticles decrease from about 20 nm to about 10 nm in mean size with the decrease of the laser power from 100 to 40 mJ/pulse. The other conditions are unchanged, and the results can be seen in Figure 4. However, the thickness of the outer wrapping carbon layer is almost unchanged (~2 nm) at different ablation powers in our case. The reasons are that the higher power laser beams induce a higher density Au plasma plume on the target surface, and higher density Au particles are more easily nucleated to form larger size nanoparticles [16]. Therefore, the size of the inner noble metal nanoparticles can be controlled by adjusting the power of laser ablation, and the controllable growth of nanoparticles can be achieved by this method.

**Figure 5:**
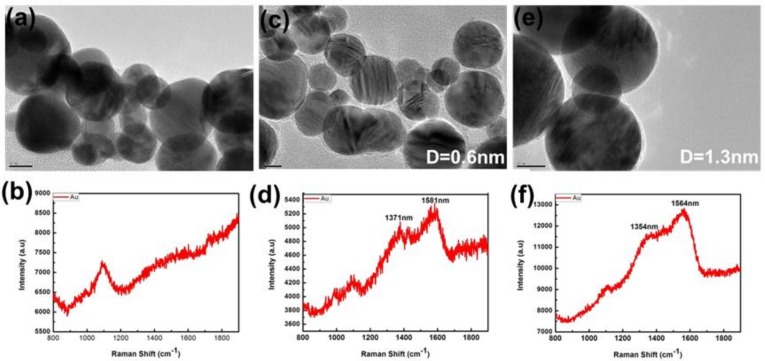
The TEM morphology characterization and Raman spectrum of the Au@C nanoparticles in different solutions. (**a**,**b**) water; (**c**,**d**) pure ethanol; and (**e**,**f**) toluene-ethanol mixed solution with the volume ratio of 1:1. The scales in (a), (e) and (f) are all 10 nm.

As expected, further experiments have revealed that the amount of the nanoparticles in the solution increases with the duration of the laser ablation from 2 to 60 min, but the thickness of the carbon shell is almost unchanged. However, the composition of liquid medium strongly influences the thickness of the outer carbon shell surrounding the inner noble metal nanoparticles, as shown in Figure 5. The higher content of the carbon component leads to the thicker carbon shell, or vice versa. In the water solution, there is no carbon shell generated, as shown in Figure 5a. The D band and G band of graphitic carbon also cannot be seen in Figure 5b. If laser ablates in the pure ethanol under the same conditions (60 mJ/pulse) as above, we can also obtain the Au@C nanoparticles but with much smaller thickness (only ~0.6 nm), as shown in Figure 5c. With the increase of the toluene content in the mixed solution, the carbon shell will get thicker and thicker. With the volume ratio of 1:1, the thickness of the outer carbon shell can reach to ~1.3 nm, as shown in Figure 5e, and ~2 nm with the volume ratio of 9:1 shown in Figure 2. The Raman spectral measurements have confirmed existence of carbon shell on the surface of noble metal nanoparticles (similar to that shown in Figure 3). Therefore, by laser ablation in different components of organic solution, we obtained different thickness of the ultrathin carbon layer, and successfully realize the effective control of the thickness of the outer carbon shell of the noble metal nanoparticles.

In addition, as shown in Figure S7, the color of the colloid solution is changed obviously in the four solutions of water, pure ethanol, toluene-ethanol mixed solution with volume ratios of 1:1 and 9:1, respectively. The color of the colloid solution of Au nanoparticles prepared in water is more transparent and red in color (A). The color is deep purple red for the product obtained in pure ethanol (B). In the toluene-ethanol mixed solution, the color is a darker purple-brown (C,D).

From the above results, the formation mechanism of the carbon-wrapped noble metal nanoparticles could be easily speculated as follows. When the laser beam irradiates on the surface of metal target, the high-pressure metal plasma will be quickly formed on the solid-liquid interface [34,35,36]. Subsequently, such metal plasma will ultrasonically and adiabatically expand, leading to cooling of the metal plume region and hence to formation of metal nanoparticles. At the same time, C-O and C-C ligands in ethanol or toluene molecules, at the interface between the metal plasma plume and the mixed solution, will be broken due to the extreme conditions to form carbon atoms. These carbon atoms would deposit on the preformed Au or Ag nanoparticles to form ultrathin carbon layer. For the laser ablation duration and power, it could be attributed to the number balance between the laser ablation-induced C and metal nanoparticles. The longer ablation duration or higher power will not only produce the more C-C broken bonds in the solvent molecules but also form the more Au or Ag nanoparticles. On the other hand, the thickness of the carbon shell surrounding the metal nanoparticles should significantly depend on the ability of liquid phase to supply carbon atoms at a certain laser fluence. Thus, some carbon-abundant solutions (such as toluene) could produce a thicker carbon layer than ethanol.

**Figure 6:**
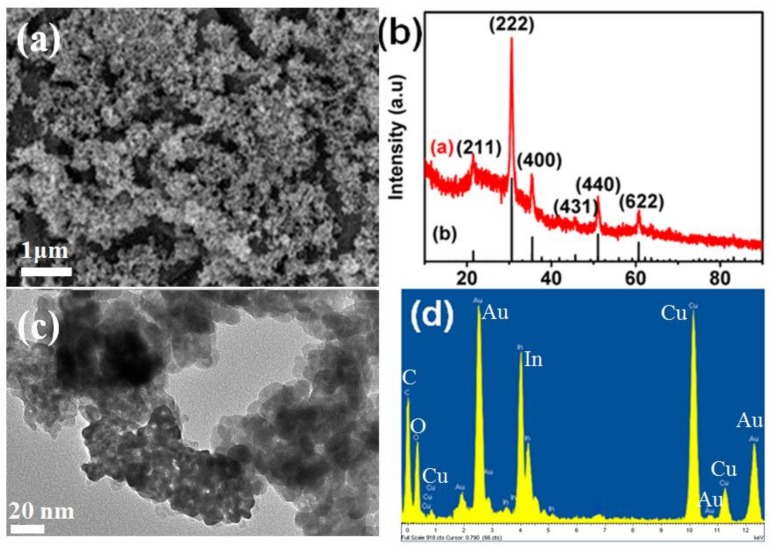
(**a**) SEM image of In_2_O_3_ thin film on ceramic tube; (**b**) corresponding XRD pattern; (**c**) TEM picture of as-prepared Au@C modified In_2_O_3_; and (**d**) its energy spectrum.

The In_2_O_3_ film is modified by Au@C nanoparticles (marked as In_2_O_3_/Au@C) with the solution impregnation method, which the Au@C nanoparticles are prepared in toluene-ethanol mixed solution with the volume ratio of 9:1 and the power of 60 mJ/pulse. From the Figure 6a, it can be seen that the In_2_O_3_ film on the ceramic tube is a dense granular film, and the main diffraction peaks of prepared In_2_O_3_ film is corresponding to cubic phase structure of In_2_O_3_ (JCPDS 74-1990) from its XRD pattern (Figure 6b). The TEM picture of the In_2_O_3_/Au@C sample and its energy spectrum can be seen in Figure 6c,d. On the surface of In_2_O_3_ film, there are many of spherical nanoparticles, which are the modifying Au@C nanoparticles. On the other hand, from the spectral peaks of Au elements, it is also proved that the Au@C nanoparticles are successfully modified on the surface of In_2_O_3_ granular film with solution impregnation method. The Au nanoparticles prepared by laser ablation in water solution are also modified on the In_2_O_3_ film (marked as In_2_O_3_/Au) with the same method.

**Figure 7:**
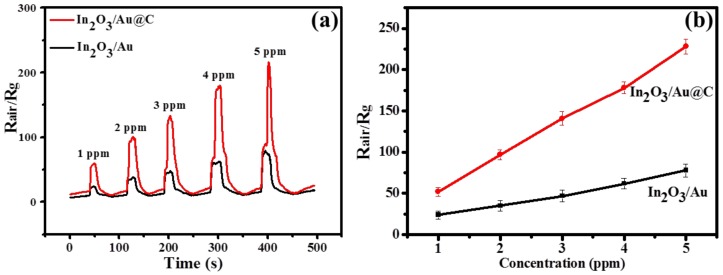
(**a**) The response curves as functions of test time to H_2_S and (**b**) the sensitivity versus H_2_S concentration of In_2_O_3_/Au@C and In_2_O_3_/Au sensor at a concentration ranging from 1–5 ppm with RH = 60% at room temperature.

**Figure 8:**
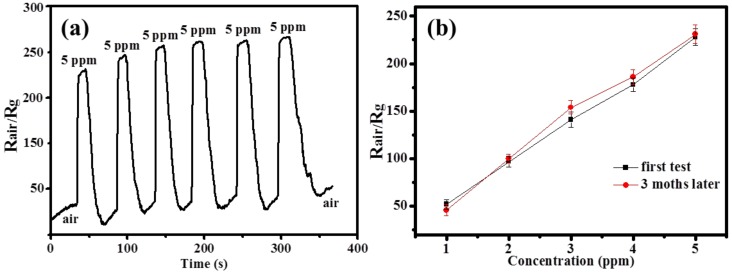
(**a**) The reproducibility response curves of In_2_O_3_/Au@C sensor to H_2_S with a concentration of 5 ppm at room temperature; and (**b**) the concentration-dependent response curve to H_2_S from 1–5 ppm at room temperature measured before and after three months for In_2_O_3_/Au@C sensor. All are tested under the ambience with 60% RH.

The gas-sensing properties of In_2_O_3_, In_2_O_3_/Au and In_2_O_3_/Au@C films to H_2_S gas are measured at room temperature from 1 to 5 ppm with a relative humidity (RH) of 60%, and the results can be seen in Figure 7 and Figure S8. For the In_2_O_3_ film at a concentration of 1 ppm, it has an excellent gas-sensitive response to H_2_S gas (about 90) at room temperature, but it cannot be recovered, as shown in Figure S8. It can be seen that for the In_2_O_3_/Au@C films the sensing sensitivity is 52, 97, 141, 178, and 228 from the concentration of 1 to 5 ppm to H_2_S with RH = 60% at room temperature, respectively. For the In_2_O_3_/Au films that is 24, 35, 47, 62, and 78 at the same conditions, respectively. Thus, the In_2_O_3_/Au@C films have a better gas sensing sensitivity to H_2_S gas than In_2_O_3_/Au films. However, the In_2_O_3_/Au films have a better response and recovery time (defined as the times to reach 90% of resistance change) shown in Figure 7a. For example, at the concentration of 4 ppm to H_2_S gas, the response and recovery time is 9 and 20 s for the In_2_O_3_/Au films, which is 16 and 33 s for the In_2_O_3_/Au@C films. That may be related to the catalytic ability of Au nanoparticles, and for the In_2_O_3_/Au films more Au nanoparticles are easily exposed to H_2_S gas, which are not coated by carbon layers. They will have a better catalytic performance and response and recovery capability. Additionally, for the In_2_O_3_/Au@C films at the concentration of 5 ppm to H_2_S gas there is a sharp response curve, but it has achieved sensor signal saturation, which can be seen in the later discussion (the reproducibility response curves of In_2_O_3_/Au@C sensor to H_2_S with a concentration of 5 ppm at room temperature in Figure 8). Similar to most semiconductor material, In_2_O_3_ is an n-type semiconducting metal oxide. When it is exposed to air, oxygen would be adsorbed on the surface of the In_2_O_3_ film and turned into chemisorbed oxygen, such as O_2_^2−^ or O_2_^−^, which plays a role as trap electrons and surface acceptors, and the resistance of the In_2_O_3_ film increases. If the In_2_O_3_ film is exposed to H_2_S gas, which is a strong reducing gas, the H_2_S molecules will react with the O_2_^2−^ or O_2_^−^ adsorbed on the surface of In_2_O_3_ film. Then the captured electrons will release back to the bulk, and the resistance of the In_2_O_3_ film decrease. Thus, the response of the samples to H_2_S gas R_air_/R_g_ will drastically increase. When the In_2_O_3_ film once is exposed to air, it will return to the initial state, and so on and so on. Exposed to high concentration of H_2_S gas, the decrease of resistance of the In_2_O_3_ film is more obvious. The Au nanoparticles and carbon shell also plays an important role in the process, and the affect mechanism will discussed in detail later. For the two films, there is also a good linear relationship with concentration of H_2_S gas as shown in Figure 7b, which is favorable to the practical application. For each concentration of H_2_S gas, the number of repeat measurements is six and the resulting standard deviation for the two films can also be seen in Figure 7b.

From the above results, it can be seen that the In_2_O_3_ films modified by Au@C have the best sensitivity. Thus, the practical performances of the In_2_O_3_/Au@C sensor are tested. In Figure 8a, it can be seen that the In_2_O_3_/Au@C gas sensor still have a similar response curves in the six cycles, and the sensing sensitivity is 237, 246, 252, 257, 258, and 261 with 60% RH to H_2_S gas with a concentration of 5 ppm at room temperature, respectively. As the number of cycles increases, the sensing sensitivity for the H_2_S gas increases as well. The reason is that at room temperature when the sample is exposed to H_2_S gas with a concentration of 5 ppm, it will lead to the decrease of resistance of the In_2_O_3_/Au@C sample and the dramatic increase of the test signal (R_air_/R_g_). When it is exposed to air, the sample will return to the initial state and the test signal will decrease. However, in this process the ultrathin carbon shell may adsorb some residual H_2_S gas, which results in the resistance of the In_2_O_3_/Au@C sample increase in air compared to the first state. As the number of cycles increases, more residual H_2_S gas may be adsorbed on the surface of ultrathin carbon shell, and the test signal also drift higher. Further, the concentration dependent response curve to H_2_S from 1–5 ppm at room temperature with 60% RH is measured after three months for the In_2_O_3_/Au@C sensor, and only a slight change happened in three months, as shown in Figure 8b. It indicates that the In_2_O_3_/Au@C sensor also has a good long-term stability. Additionally, the resulting standard deviation is shown in Figure 8b and the number of repeat measurements is six for each concentration of H_2_S gas.

**Figure 9:**
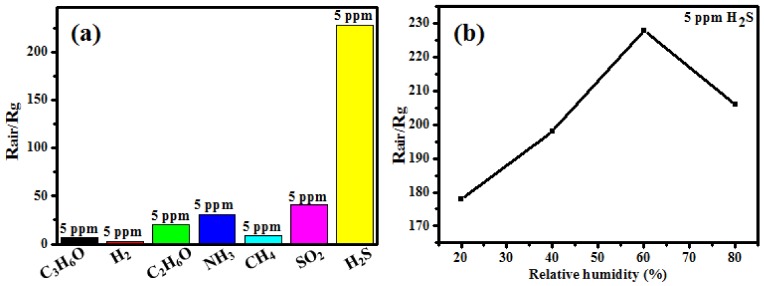
(**a**) Some potential interferential gases for the In_2_O_3_/Au@C sensor at room temperature with 60% RH compared to H_2_S; and (**b**) the steady response curve to H_2_S with a concentration of 5 ppm the at room temperature as a function of the ambient humidity from 20 to 80%.

The selectivity is very important for a gas sensor, so the In_2_O_3_/Au@C sensor is exposed to six other kinds of oxidizing or reducing interferential gases at room temperature with 60% RH, such as C_3_H_6_O, H_2_, C_2_H_6_O, NH_3_, CH_4_, and SO_2_, respectively. As shown in Figure 9a, the sensing sensitivity to the seven kinds of gases is 7, 3, 20, 31, 9, 41, and 228, respectively, the concentration of which is 5 ppm, including H_2_S gas. It can be seen that the sensitivity to H_2_S gas is highest, which is several or more than one hundred times that of other gases. As the consequence, the In_2_O_3_/Au@C sensor will have an excellent selective gas sensing to H_2_S in the real environment. Further, the response curves to H_2_S with different RH values at a concentration of 5 ppm are shown in Figure 9b at room temperature, and the sensing sensitivity is 177, 198, 228, and 206, respectively. The response curve to H_2_S increases to the maxima with rise of the ambient RH value up to 60%, and they decreases at a higher RH. Obviously, with 60% RH the In_2_O_3_/Au@C sensor has the best response ability.

From the above results, it is known that the Au and Au@C nanoparticles can improve the performance of In_2_O_3_ gas sensors. This is due to the electronic sensitization mechanism and chemical sensitization mechanism of nanoparticles, and the nanoparticles prepared by laser ablation of the metal targets in liquids have a large number of defects and chemical dangling bonds in the inner and on the surface of nanoparticles, which have higher activity [15]. On the one hand, the nanoparticles can enhance the electron density on the surface of the In_2_O_3_ film and adjust the resistance by the electronic sensitization mechanism. On the other hand, they have higher catalytic activity by the chemical sensitization mechanism and the spillover effect in the catalysis process [37]. They may provide more active sites for the adsorption of molecular oxygen and tracer gas. Then more electrons are provided for the redox reaction occur on the surface of In_2_O_3_ film, and the speed of gas sensitive reaction is accelerated. In addition, it is also observed that the In_2_O_3_ film modified by Au@C nanoparticles has better gas sensing properties than that modified by Au nanoparticles, indicating that the ultrathin carbon layer plays an important role on the gas sensing process. This is due to the unique electronic properties of carbon materials, such as high carrier mobility and the high sensitivity to the changes of resistance [38,39,40]. The ultrathin carbon layer can rapidly transform the electric carriers generated from the sensing process, and local p-n heterojunctions are created between carbon layer and In_2_O_3_ film, in which the carbon layer plays a role as a p-type semiconductor [37]. As a results, the performance of gas sensor is enhanced more. From the HRTEM of Au@C sample (Figure 1), it can be seen that the outer ultrathin carbon layer is about 2 nm in thickness with several carbon shells, which cannot prevent the contact of the analyte gas with the Au nanoparticles. The analyte gas can pierce through the outer carbon shells easily [41]. Finally, the more specific impact mechanisms will be studied in detail in future investigations.

## 4. Conclusions

In summary, the noble metal nanoparticles wrapped by an ultrathin carbon layer were prepared in one step based on laser ablation of the metal targets in the carbon-containing solutions. Laser irradiation of the targets not only formed the metal plasma instantly, but also the carbon atoms by breaking C-O and C-C ligands in the solution, which led to the formation of metal nanoparticles and subsequent carbon-wrapping. The thickness of the wrapped carbon layer could be tuned and controlled mainly by the carbon content in solutions, and it would reduce to ~1.3 nm by changing the proportion of toluene in the ethanol-toluene mixed solution. The In_2_O_3_ film modified by Au@C nanoparticles shows better gas sensitivity performance to H_2_S gas from 1 to 5 ppm at room temperature, and the ultrathin carbon layer plays an important role on the gas sensing process. This study could also be suitable for the preparation of other metal nanoparticles with wrapped ultrathin carbon layers.

## Supplementary Materials

The following are available online at http://www.mdpi.com/2072-666X/9/6/278/s1, Figure S1: The energy spectrum of Au@C nanoparticles products by ablation of Au target in the toluene-ethanol solution with the volume ratio 9:1, Figure S2: TEM image of products induced by ablation of Au target in the water and the inset: selected area electronic diffraction of the particles, showing formation of Au nanoparticles with the size below 20 nm, Figure S3: TEM image of products induced by ablation of Ag target in the water, Figure S4: The products induced by ablation of Ag target in the toluene-ethanol solution with the volume ratio 9:1. (a): TEM image. (b): particle size distribution (data from (a)). (c): The local magnified image of (a). The inset is the selected area electronic diffraction, Figure S5: The energy spectrum of Ag@C nanoparticles products by ablation of Ag target in the toluene-ethanol solution with the volume ratio 9:1, Figure S6: The gravimetric analysis curve of the Au@C nanoparticles sample, Figure S7: The colour of the colloid solution obtained in the four solutions of (A) water; (B) pure ethanol; (C) toluene-ethanol mixed solution with the volume ratio of 1:1; and (D) toluene-ethanol mixed solution with the volume ratio of 9:1, Figure S8: The response curve (Rair/Rg) as function of test time to H2S gas with 1 ppm concentration at room temperature
