# Supplementary material for: The Fabrication of Au@C Core/Shell Nanoparticles by Laser Ablation in Solutions and Their Enhancements to a Gas Sensor
Source: Micromachines (Basel). 2018 Jun 1;9(6):278. doi: 10.3390/mi9060278 (PMC6187519; doi:10.3390/mi9060278)
Supplement: Supplementary file 1 [file micromachines-09-00278-s001.pdf]

The Fabrication of Au@C Core/Shell Nanoparticles by Laser Ablation in Solutions and their Enhancements to a Gas Sensor

Xiaoxia Xu†, Lei Gao†, Guotao Duan*

Key Laboratory of Materials Physics, Anhui Key Laboratory of Nanomaterials and Nanotechnology, Institute of Solid State Physics, Chinese Academy of Sciences, Hefei 230031, P. R. China

* Correspondence: duangt@issp.ac.cn

† These authors contributed equally to this work.

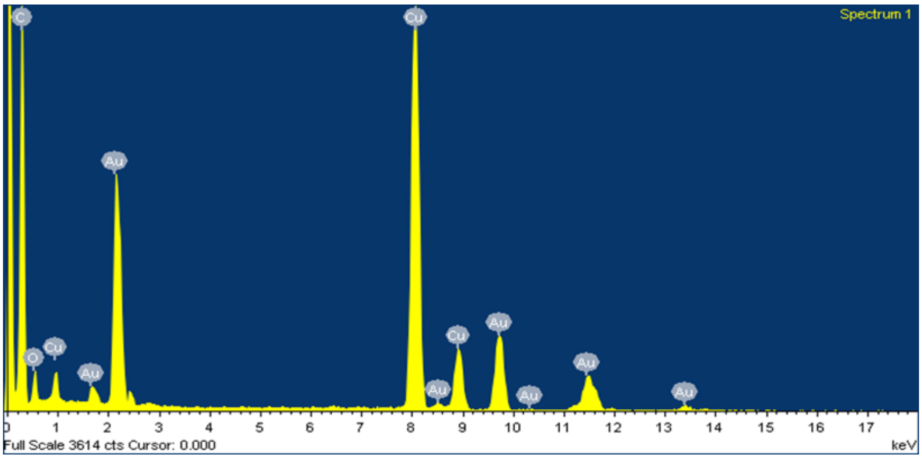

Figure S1. The energy spectrum of Au@C nanoparticles products by ablation of Au target in the toluene-ethanol solution with the volume ratio 9:1.

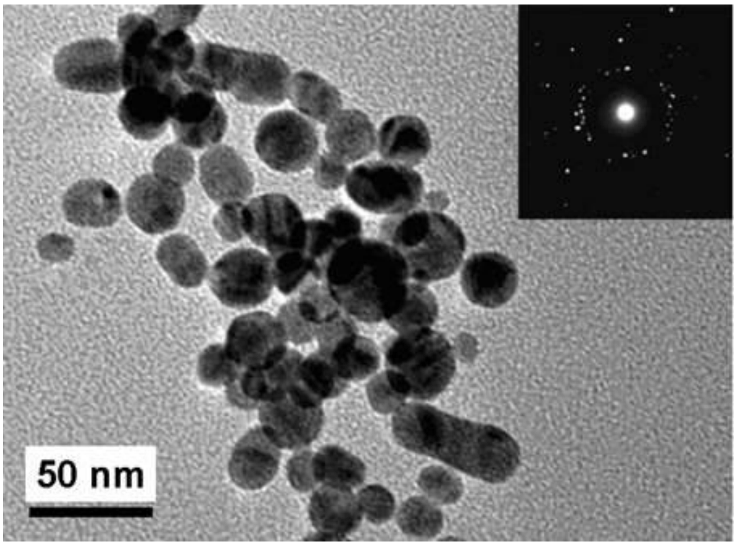

Figure S2. TEM image of products induced by ablation of Au target in the water and the inset: selected area electronic diffraction of the particles, showing formation of Au nanoparticles with the size below 20 nm.

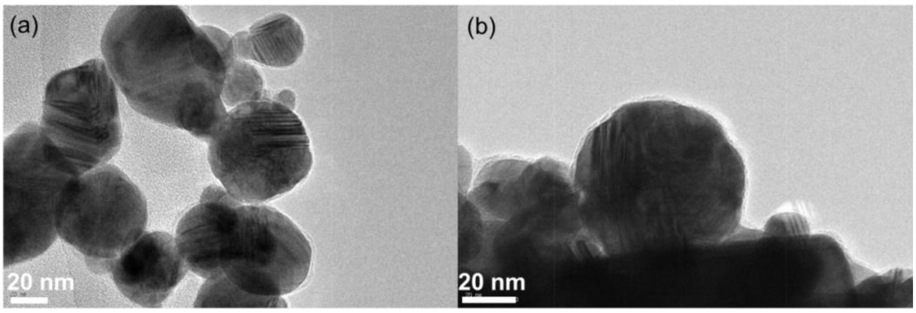

Figure S3. TEM image of products induced by ablation of Ag target in the water.

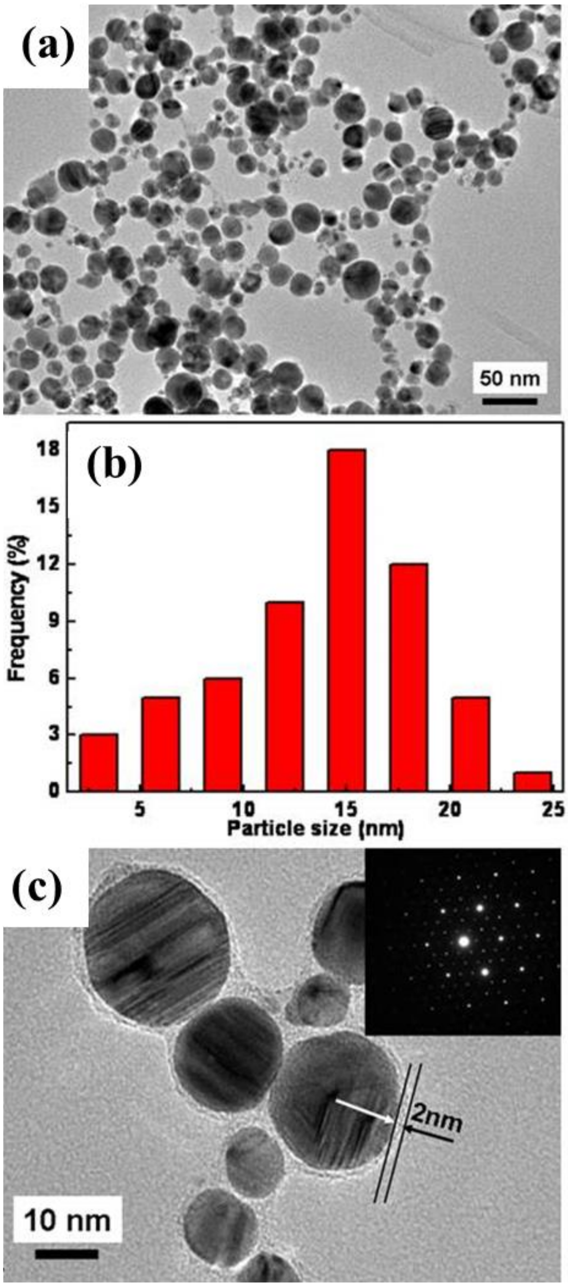

Figure S4. The products induced by ablation of Ag target in the toluene-ethanol solution with the volume ratio 9:1. (a): TEM image. (b): particle size distribution (data from (a)). (c): The local magnified image of (a). The inset is the selected area electronic diffraction.

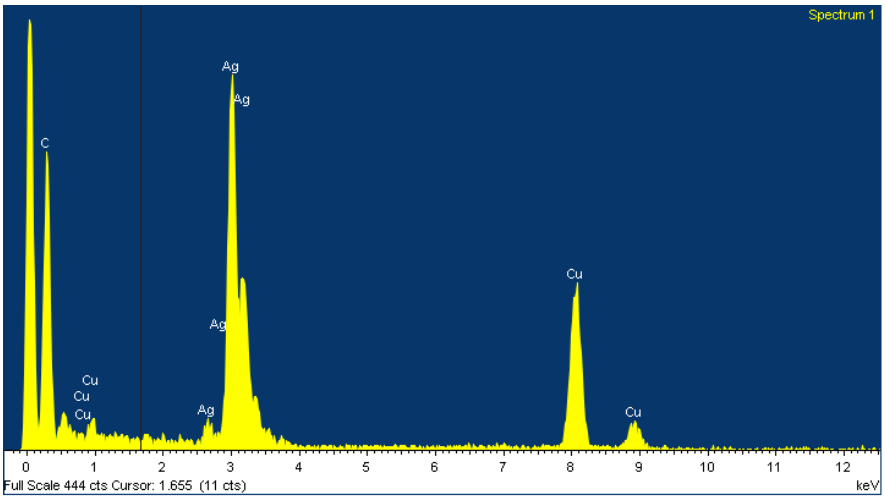

Figure S5. The energy spectrum of Ag@C nanoparticles products by ablation of Ag target in the toluene-ethanol solution with the volume ratio 9:1.

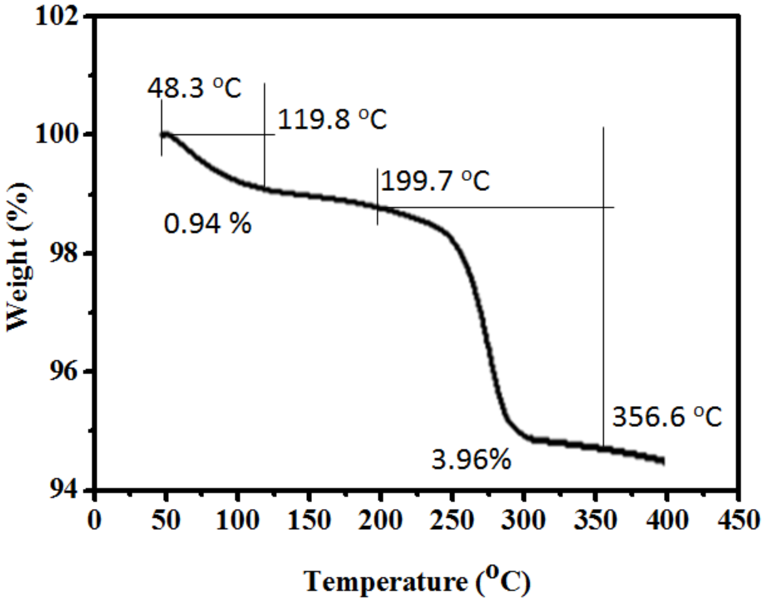

Figure S6. The gravimetric analysis curve of the Au@C nanoparticles sample.

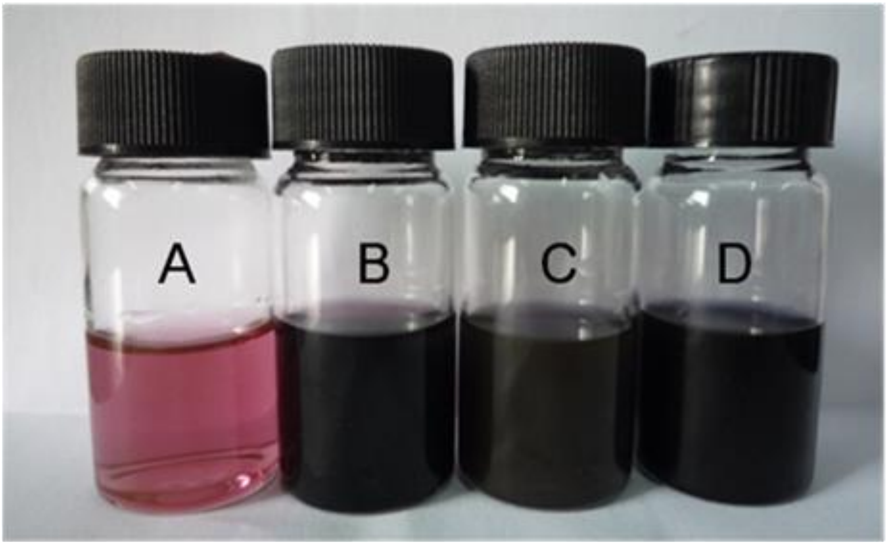

Figure S7. The colour of the colloid solution obtained in the four solutions of (A) water; (B) pure ethanol; (C) toluene-ethanol mixed solution with the volume ratio of 1:1; and (D) toluene-ethanol mixed solution with the volume ratio of 9:1.

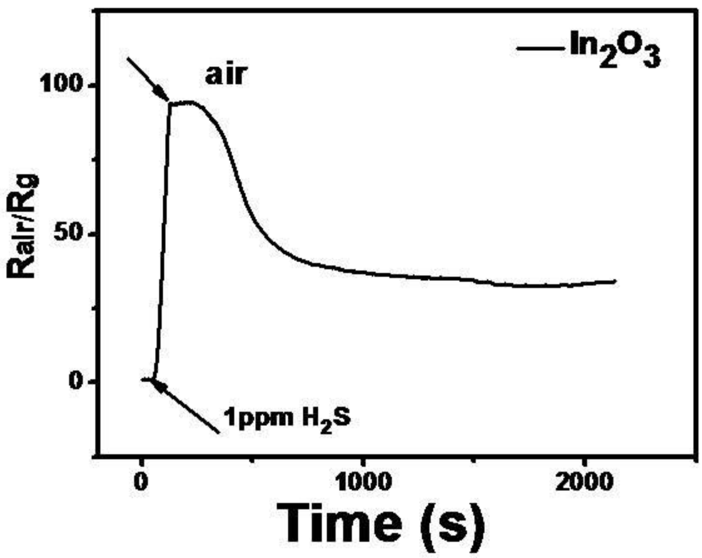

Figure S8. The response curve (R_{air}/R_g) as function of test time to H_2S gas with 1 ppm concentration at room temperature.
